# Climate Classification System–Based Determination of Temperate Climate Detection of *Cryptococcus gattii* sensu lato

**DOI:** 10.3201/eid2509.181884

**Published:** 2019-09

**Authors:** Emily S. Acheson, Eleni Galanis, Karen Bartlett, Brian Klinkenberg

**Affiliations:** University of British Columbia, Vancouver, British Columbia, Canada (E.S. Acheson, E. Galanis, K. Bartlett, B. Klinkenberg);; British Columbia Centre for Disease Control, Vancouver (E. Galanis)

**Keywords:** climate change, classification, climate, disease, ecology, fungi, Vancouver Island, Canada, temperate climate, *Cryptococcus gattii*, Köppen-Geiger system, solar definition, emerging infections

## Abstract

We compared 2 climate classification systems describing georeferenced environmental *Cryptococcus gattii* sensu lato isolations occurring during 1989–2016. Each system suggests the fungus was isolated in temperate climates before the 1999 outbreak on Vancouver Island, British Columbia, Canada. However, the Köppen-Geiger system is more precise and should be used to define climates where pathogens are detected.

A global systematic framework is needed to define climates where pathogens are detected. The 1999 cryptococcal outbreak of the fungal species complex *Cryptococcus gattii* sensu lato on Vancouver Island (*1*), British Columbia, Canada, was described as the first temperate climate emergence of the pathogen because, before this event, *C. gattii* s.l. was largely reported in areas described as tropical and subtropical (Appendix Figure). This assumption led to the belief that the Vancouver Island outbreak might be associated with a changing climate. The lack of precision and standardization of climate classification in the health literature makes comparing emergence areas around the world and determining why or how the organism emerged in that area difficult.

The lack of consensus might largely be rooted in the lack of a global systematic framework to define climates where pathogens are detected. Specifically, a standardized definition of tropical, subtropical, and temperate to compare pathogen isolation areas worldwide is unavailable. Here, we compare the 2 solar climate definitions and the Köppen-Geiger climate classification system to determine whether the 1999 Vancouver Island outbreak was the first-ever detection of *C. gattii* s.l. in a temperate environment and which system should be used for a global systematic climate classification framework.

## The Study

We used environmental isolations of *C. gattii* s.l. (i.e., detections in plant and soil samples) to map global distribution. Geographically defined human and animal records of *C. gattii* infection are not always accessible because of privacy restrictions. In addition, the dates and locations of *C. gattii* s.l. exposures are often uncertain because of the mobility of animals and humans and *C. gattii*’s long, undetermined incubation and latency periods (*2*). We extended the data of a database of globally georeferenced environmental isolations of *C. gattii* s.l. from the peer-reviewed literature (*3*) through November 2018 (Appendix Table). We excluded studies in which only the country of sampling was specified because many countries extend through multiple climates. We recorded the earliest year of isolation or, if a sampling year was not specified, the year of study publication. In total, we used 83 geographically unique coordinates of *C. gattii* isolations occurring during 1989–2016.

According to the solar definition (the predominant definition used in the *C. gattii* literature to describe isolation climates), tropical, subtropical, and temperate regions are denoted by latitudinal boundaries (*4*). In contrast, the Köppen-Geiger system (*5*) uses precipitation, temperature, and vegetation traits to produce 5 main climate groups (tropical or equatorial, arid, temperate, continental, and polar) and subgroups and is the most widely used climate classification system by researchers, including medical geographers, worldwide (*6*). For the solar definition of climate, we set the latitudinal boundaries of the tropics at 23.4 degrees north and south of the equator, the area of the subtropics as the tropical extent to 35 or 40 degrees north and south of the equator, and the temperate area as the subtropical extent to 66.5 degrees north and south of the equator (*4*,*7*). For the Köppen-Geiger system, we used a map depicting the climate characteristics observed during 1976–2000 with a spatial resolution of 0.5 degrees (http://koeppen-geiger.vu-wien.ac.at/shifts.htm). Using ArcMap version 10.5.1 (ESRI 2017, https://www.esri.com), we overlaid the isolation coordinates of *C. gattii* on each map and extracted the corresponding climate classifications. If exact coordinates of the sample were not specified, we used ArcMap to estimate coordinates for the centroid of the park, city, or town where the sampling occurred. We first compared the climates assigned to isolations by classification system and then determined which positive sampling years had >1 isolation in a temperate region and how many of these years preceded 1999.

**Figure 1:**
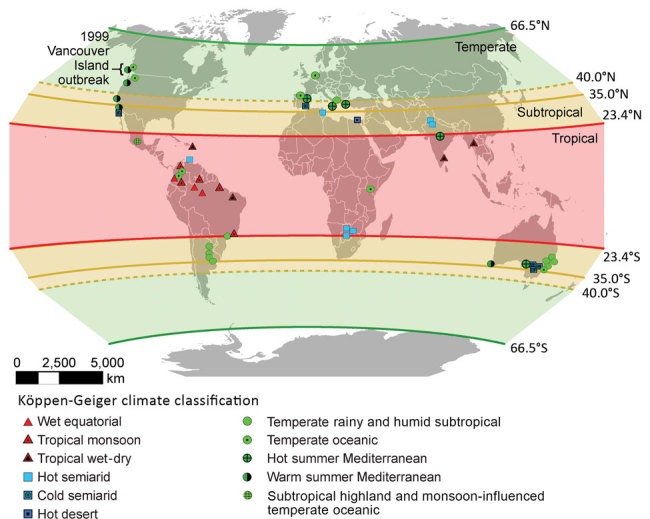
Global environmental isolations of *Cryptococcus gattii* sensu lato, 1989–2016. We mapped 83 unique geographic coordinates of *C. gattii* s.l. isolations and labeled them according to their Köppen-Geiger climate classification. Overlapping symbols of the same Köppen-Geiger climate classification (where isolations were 0–200 km apart) were removed for easier visualization. The solar definition of the tropics is shown as the semitransparent red area extending from the equator to 23.4 degrees north and south of the equator, the subtropics as the yellow area extending from the tropics to either 35 (solid line) or 40 (dashed line) degrees north and south of the equator, and the temperate zone as the green area extending from the subtropics to 66.5 degrees north and south of the equator.

**Figure 2:**
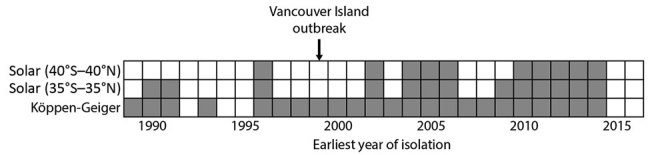
Timeline of environmental *Cryptococcus gattii* sensu lato isolations in temperate climates, by climate definition, 1989–2016. Gray squares indicate years in which >1 isolate from a temperate climate was obtained, and white squares indicate years in which no such isolate was obtained.

The solar definition classified the 83 environmental *C. gattii* s.l. isolations as tropical, subtropical, or temperate (Figure 1). By comparison, the Köppen-Geiger system classified these same isolations into 11 different climate subgroups (Figure 1). Both systems identified >1 temperate-climate environmental *C. gattii* isolation (*8*) before 1999 (Figure 2).

Both variations of the solar definition and the Köppen-Geiger system classified the environmental samples of *C. gattii* s.l. isolated on Vancouver Island during the outbreak as temperate (Appendix Table). According to the Köppen-Geiger system, the Vancouver Island outbreak areas have 2 different types of temperate climates: temperate oceanic and warm summer Mediterranean, that is, precipitation conditions that range from dry summers to fully humid year-round with warm summer temperatures (*5*). According to the more restrictive solar definition of temperate, the environmental *C. gattii* s.l. isolation coordinates from only 1 year before 1999 could be classified as temperate (Figure 2). By contrast, the Köppen-Geiger system classified the coordinates of environmental isolations from 7 different years before 1999 as temperate (Figure 2; Appendix Table). These isolation coordinates included areas in California (*9*,*10*) and southwestern and southeastern Australia (*9*,*11*). The solar definition of climate largely categorized these areas as tropical or subtropical (Appendix Table), but the Köppen-Geiger system labeled them as temperate oceanic and warm summer Mediterranean.

## Conclusions

Both versions of the solar definition and the Köppen-Geiger system suggest that the Vancouver Island outbreak was not the first-ever temperate detection of *C. gattii* s.l. However, in terms of geographic scale, the solar definition of tropical, subtropical, and temperate are too coarse for the purposes of classifying or describing areas of local clinical, veterinary, or environmental isolations of *C. gattii* s.l. or any other environmentally contracted pathogen. Species, including pathogenic species, can live within geographically smaller refugia that maintain their climatic and biological needs across larger landscapes and solar boundaries, depending on topography, microclimates, and habitat fragmentation (*12*,*13*). Although the Köppen-Geiger system still generalizes across precipitation, temperature, and vegetation, the system accounts for more environmental variation and provides temperature and precipitation limits for each climate subtype. The Köppen-Geiger system is the most widely used climate classification system worldwide (*5*) and also provides projected maps for future climate shifts, making this system ideal as a global systematic framework for tracking the climates of pathogen detection. We, therefore, propose the use of the Köppen-Geiger system, as opposed to either of the overgeneralized solar definitions, for the sake of precision and consistency across global records when characterizing pathogen detection areas.

One limitation of our study was dependence on the reporting of environmental *C. gattii* samples in the English language peer-reviewed literature. As a result, our findings are an underrepresentation of the full global extent of *C. gattii* s.l. in the environment. Other evidence exists for the emergence of *C. gattii* s.l. in temperate climates before 1999. For example, in addition to the environmental isolations made in Busselton, Western Australia, Australia (*9*), in 1993, multiple *C. gattii* infections in animals were reported in southwestern Australia, including Perth, before 1999 (*14*). Both Busselton and Perth fall within a temperate Köppen-Geiger climate (*5*) (Figure 1). Another limitation was variability in the descriptions of pathogen detection areas. For example, some studies provided the exact coordinates of *C. gattii* sampling, and others provided a park or city name. Providing the exact coordinates offers the greatest certainty of a detection location and better precision in climate classification.

By using *C. gattii* s.l. as an example for mapping georeferenced pathogen isolations worldwide, we demonstrated the opportunity to improve pathogen monitoring through the development of a standardized global climate classification framework. Using more spatially specific climate classification methods, such as the Köppen-Geiger system used by medical geographers, coupled with the continued reporting of pathogen isolation locations, will improve comparability of pathogen detection in new natural environments.

Appendix Additional information for determining the temperate climate detection of *Cryptococcus gattii* sensu lato using a climate classification system–based approach.
