# Supplementary material for: Climate Classification System–Based Determination of Temperate Climate Detection of Cryptococcus gattii sensu lato
Source: Emerg Infect Dis. 2019 Sep;25(9):1723–6. doi: 10.3201/eid2509.181884 (PMC6711209; doi:10.3201/eid2509.181884)
Supplement: Appendix — Additional information for determining the temperate climate detection of Cryptococcus gattii sensu lato using a climate classification system–based approach. [file 18-1884-Techapp-s1.pdf]

Article DOI: https://doi.org/10.3201/eid2509.181884

Climate Classification System–Based Determination of Temperate Climate Detection of *Cryptococcus gattii* sensu lato

Appendix

Methods for Updated Literature Review

To assess how historical (i.e., pre-1999) areas of *C. gattii* endemicity have been described in the literature, we searched for peer-reviewed English articles published from January 1970 through November 2018 that directly analyzed or reviewed the ecology and/or geographic distribution of *C. gattii*. We searched Google Scholar (http://scholar.google.com), ScienceDirect (http://www.sciencedirect.com), and Web of Science (http://thomsonreuters.com/thomson-reuters-web-of-science). For species, we selected the inputs "either/or" for "*Cryptococcus gattii*," "*Cryptococcus neoformans* var. *gattii*," or "*Cryptococcus bacillisporus*" in the search engine, where the last was considered synonymous with *C. gattii* in the late 1970s and early 1980s (*1*); for ecologic or geographic studies, we selected "either/or" for "ecolog*," "geograph*," "distribution," and "niche." In addition to assessing how historical areas of endemicity of *C. gattii* were described, we also identified which studies were used as the original sources of these statements.

We originally found 105 articles that studied *C. gattii* ecology or geographic distributions, but further narrowed our results down to 73 articles that directly discussed areas of *C. gattii* endemicity before the Vancouver Island outbreak. Articles were classed as "restricted" if they worded *C. gattii* distributions as "restricted to," "only found in," or "confined to" (sub)tropical areas, or variations thereof. Articles were classed as "general" if they worded *C. gattii* distributions as "mainly," "predominantly," or "primarily" in (sub)tropical areas, or variations thereof.

Of the 73 articles reviewed, 35 studies contained statements claiming *C. gattii* was restricted to the (sub)tropics before the Vancouver Island outbreak (i.e., "restricted" statements),

while 38 studies also acknowledged other historical areas of endemicity outside the (sub)tropics (i.e., "general" statements) (Appendix Figure). The same studies were often cited as the sources of both statements. For example, 2 seminal papers published in 1984 on global *Cryptococcus neoformans* and *C. gattii* distributions (*2*,*3*) were primarily or secondarily cited in 24 of the 35 studies making "restricted" statements but were also cited in 10 of the 38 studies making "general" statements. The authors demonstrated a higher prevalence of *C. gattii* in (sub)tropical areas compared to temperate ones but stated the fungus was not entirely restricted to these warmer areas.

References

1. Kwon-Chung KJ, Bennett JE, Theodore TS. *Cryptococcus bacillisporus* sp. nov.: serotype B-C of *Cryptococcus neoformans*. Int J Syst Evol Microbiol. 1978;28:616–20. https://doi.org/10.1099/00207713-28-4-616

2. Kwon-Chung KJ, Bennett JE. Epidemiologic differences between the two varieties of *Cryptococcus neoformans*. Am J Epidemiol. 1984;120:123–30. PubMed https://doi.org/10.1093/oxfordjournals.aje.a113861

3. Kwon-Chung KJ, Bennett JE. High prevalence of *Cryptococcus neoformans* var. *gattii* in tropical and subtropical regions. Zentralbl Bakteriol Mikrobiol Hyg A. 1984;257:213–8. PubMed

4. Ellis DH, Pfeiffer TJ. Natural habitat of *Cryptococcus neoformans* var. *gattii*. J Clin Microbiol. 1990;28:1642–4. PubMed

5. Sorrell TC, Chen SCA, Ruma P, Meyer W, Pfeiffer TJ, Ellis DH, et al. Concordance of clinical and environmental isolates of *Cryptococcus neoformans* var. *gattii* by random amplification of polymorphic DNA analysis and PCR fingerprinting. J Clin Microbiol. 1996;34:1253–60. PubMed

6. Halliday CL, Bui T, Krockenberger M, Malik R, Ellis DH, Carter DA. Presence of α and **a** mating types in environmental and clinical collections of *Cryptococcus neoformans* var. *gattii* strains from Australia. J Clin Microbiol. 1999;37:2920–6. PubMed

7. Pfeiffer T, Ellis D. Environmental isolation of *Cryptococcus neoformans gattii* from California. J Infect Dis. 1991;163:929–30. PubMed https://doi.org/10.1093/infdis/163.4.929

8. Pfeiffer TJ, Ellis DH. Environmental isolation of *Cryptococcus neoformans* var. *gattii* from *Eucalyptus tereticornis*. J Med Vet Mycol. 1992;30:407–8. PubMed https://doi.org/10.1080/02681219280000541

9. Lazera MS, Wanke B, Nishikawa MM. Isolation of both varieties of *Cryptococcus neoformans* from saprophytic sources in the city of Rio de Janeiro, Brazil. J Med Vet Mycol. 1993;31:449–54. https://doi.org/10.1080/02681219380000581

10. Lazéra MS, Cavalcanti MAS, Trilles L, Nishikawa MM, Wanke B. *Cryptococcus neoformans* var. *gattii*—evidence for a natural habitat related to decaying wood in a pottery tree hollow. Med Mycol. 1998;36:119–22. PubMed

11. Lazera MS, Salmito Cavalcanti MA, Londero AT, Trilles L, Nishikawa MM, Wanke B. Possible primary ecological niche of *Cryptococcus neoformans*. Med Mycol. 2000;38:379–83. PubMed https://doi.org/10.1080/mmy.38.5.379.383

12. Chakrabarti A, Jatana M, Kumar P, Chatha L, Kaushal A, Padhye AA. Isolation of *Cryptococcus neoformans* var. *gattii* from *Eucalyptus camaldulensis* in India. J Clin Microbiol. 1997;35:3340–2. PubMed

13. Montenegro H, Paula CR. Environmental isolation of *Cryptococcus neoformans* var. *gattii* and *C. neoformans* var. *neoformans* in the city of São Paulo, Brazil. Med Mycol. 2000;38:385–90. PubMed https://doi.org/10.1080/mmy.38.5.385.390

14. Montagna MT, Viviani MA, Pulito A, Aralla C, Tortorano AM, Fiore L, et al. *Cryptococcus neoformans* var. *gattii* in Italy. Note II. Environment investigation related to an autochtonous clinical case in Apulia. J Mycol Med. 1997;7:93–6.

15. Krockenberger MB, Canfield PJ, Malik R. *Cryptococcus neoformans* in the koala (*Phascolarctos cinereus*): colonization by *C. n.* var. *gattii* and investigation of environmental sources. Med Mycol. 2002;40:263–72. PubMed https://doi.org/10.1080/mmy.40.3.263.272

16. Callejas A, Ordoñez N, Rodriguez MC, Castañeda E. First isolation of *Cryptococcus neoformans* var. *gattii*, serotype C, from the environment in Colombia. Med Mycol. 1998;36:341–4. PubMed https://doi.org/10.1080/02681219880000531

17. Fortes ST, Lazéra MS, Nishikawa MM, Macedo RCL, Wanke B. First isolation of *Cryptococcus neoformans* var. *gattii* from a native jungle tree in the Brazilian Amazon rainforest. Mycoses. 2001;44:137–40. PubMed https://doi.org/10.1046/j.1439-0507.2001.00651.x

18. Mahmoud YAG. First environmental isolation of *Cryptococcus neoformans* var. *neoformans* and var. *gatti* from the Gharbia Governorate, Egypt. Mycopathologia. 1999;148:83–6. PubMed https://doi.org/10.1023/A:1007166818993

19. Gugnani HC, Mitchell TG, Litvintseva AP, Lengeler KB, Heitman J, Kumar A, et al. Isolation of *Cryptococcus gattii* and *Cryptococcus neoformans* var. *grubii* from the flowers and bark of *Eucalyptus* trees in India. Med Mycol. 2005;43:565–9. PubMed https://doi.org/10.1080/13693780500160785

20. Argüero Licea B, Garza Garza D, Flores Urbieta V, Cervantes Olivares RA. Isolation and characterization of *Cryptococcus neoformans* var. *gattii* from samples of *Eucalyptus camaldulensis* in Mexico city [in Spanish]. Rev Iberoam Micol. 1999;16:40–2. PubMed

21. Vilcins I, Krockenberger M, Agus H, Carter D. Environmental sampling for *Cryptococcus neoformans* var. *gattii* from the Blue Mountains National Park, Sydney, Australia. Med Mycol. 2002;40:53–60. PubMed https://doi.org/10.1080/mmy.40.1.53.60

22. Davel G, Abrantes R, Brudny M, Córdoba S, Rodero L, Canteros CE, et al. First environmental isolation of *Cryptococcus neoformans* var. *gattii* in Argentina [in Spanish]. Rev Argent Microbiol. 2003;35:110–2. PubMed

23. Refojo N, Perrotta D, Brudny M, Abrantes R, Hevia AI, Davel G. Isolation of *Cryptococcus neoformans* and *Cryptococcus gattii* from trunk hollows of living trees in Buenos Aires City, Argentina. Med Mycol. 2009;47:177–84. PubMed https://doi.org/10.1080/13693780802227290

24. Kidd SE, Chow Y, Mak S, Bach PJ, Chen H, Hingston AO, et al. Characterization of environmental sources of the human and animal pathogen *Cryptococcus gattii* in British Columbia, Canada, and the Pacific Northwest of the United States. Appl Environ Microbiol. 2007;73:1433–43. PubMed https://doi.org/10.1128/AEM.01330-06

25. Kidd SE, Hagen F, Tscharke RL, Huynh M, Bartlett KH, Fyfe M, et al. A rare genotype of *Cryptococcus gattii* caused the cryptococcosis outbreak on Vancouver Island (British Columbia, Canada). Proc Natl Acad Sci U S A. 2004;101:17258–63. PubMed http://dx.doi.org/10.1073/pnas.0402981101

26. Escandón P, Sánchez A, Martínez M, Meyer W, Castañeda E. Molecular epidemiology of clinical and environmental isolates of the *Cryptococcus neoformans* species complex reveals a high genetic diversity and the presence of the molecular type VGII mating type a in Colombia. FEMS Yeast Res. 2006;6:625–35. PubMed https://doi.org/10.1111/j.1567-1364.2006.00055.x

27. Nawange SR, Shakya K, Naidu J, Singh SM, Jharia N, Garg S. Decayed wood inside hollow trunks of living trees of *Tamarindus indica*, *Syzygium cumini*, and *Mangifera indica* as natural habitat of

Cryptococcus neoformans and their serotypes in Jabalpur City of Central India. J Mycol Med. 2006;16:63–71. https://doi.org/10.1016/j.mycmed.2006.02.004

28. Kidd SE, Sorrell TC, Meyer W. Isolation of two molecular types of *Cryptococcus neoformans* var. *gattii* from insect frass. Med Mycol. 2003;41:171–6. PubMed https://doi.org/10.1080/714858208

29. Escandón P, Quintero E, Granados D, Huérfano S, Ruiz A, Castañeda E. Isolation of *Cryptococcus gattii* serotype B from detritus of *Eucalyptus* trees in Colombia [in Spanish]. Biomedica. 2005;25:390–7. PubMed https://doi.org/10.7705/biomedica.v25i3.1363

30. Kidd SE, Guo H, Bartlett KH, Xu J, Kronstad JW. Comparative gene genealogies indicate that two clonal lineages of *Cryptococcus gattii* in British Columbia resemble strains from other geographical areas. Eukaryot Cell. 2005;4:1629–38. PubMed https://doi.org/10.1128/EC.4.10.1629-1638.2005

31. Mazza M, Refojo N, Bosco-Borgeat ME, Taverna CG, Trovero AC, Rogé A, et al. *Cryptococcus gattii* in urban trees from cities in North-eastern Argentina. Mycoses. 2013;56:646–50. PubMed https://doi.org/10.1111/myc.12084

32. Barbosa GG, Trilles L, Wanke B, Lazera MD. *Cryptococcus gattii* VGI and *Cryptococcus neoformans* VNI associated with wood decay in *Ficus* hollow trees in Rio de Janeiro, Brazil. Br Microbiol Res J. 2013;3:106–15. https://doi.org/10.9734/BMRJ/2013/2682

33. Firacative C, Torres G, Rodríguez MC, Escandón P. First environmental isolation of *Cryptococcus gattii* serotype B, from Cúcuta, Colombia [in Spanish]. Biomedica. 2011;31:118–23. PubMed https://doi.org/10.7705/biomedica.v31i1.342

34. Costa SP, Lazéra MS, Santos WRA, Morales BP, Bezerra CCF, Nishikawa MM, et al. First isolation of *Cryptococcus gattii* molecular type VGII and *Cryptococcus neoformans* molecular type VNI from environmental sources in the city of Belém, Pará, Brazil. Mem Inst Oswaldo Cruz. 2009;104:662–4. PubMed https://doi.org/10.1590/S0074-02762009000400023

35. Girish Kumar CP, Prabu D, Mitani H, Mikami Y, Menon T. Environmental isolation of *Cryptococcus neoformans* and *Cryptococcus gattii* from living trees in Guindy National Park, Chennai, South India. Mycoses. 2010;53:262–4. PubMed https://doi.org/10.1111/j.1439-0507.2009.01699.x

36. Romeo O, Scordino F, Criseo G. Environmental isolation of *Cryptococcus gattii* serotype B, VGI/MATα strains in southern Italy. Mycopathologia. 2011;171:423–30. PubMed https://doi.org/10.1007/s11046-010-9389-z

37. Loperena-Alvarez Y, Ren P, Li X, Schoonmaker-Bopp DJ, Ruiz A, Chaturvedi V, et al. Genotypic characterization of environmental isolates of *Cryptococcus gattii* from Puerto Rico. Mycopathologia. 2010;170:279–85. PubMed https://doi.org/10.1007/s11046-010-9296-3

38. DeBess E, Lockhart SR, Iqbal N, Cieslak PR. Isolation of *Cryptococcus gattii* from Oregon soil and tree bark, 2010-2011. BMC Microbiol. 2014;14:323. PubMed https://doi.org/10.1186/s12866-014-0323-2

39. Chowdhary A, Randhawa HS, Boekhout T, Hagen F, Klaassen CH, Meis JF. Temperate climate niche for *Cryptococcus gattii* in Northern Europe. Emerg Infect Dis. 2012;18:172–4. PubMed https://doi.org/10.3201/eid1801.111190

40. Mseddi F, Sellami A, Jarboui MA, Sellami H, Makni F, Ayadi A. First environmental isolations of *Cryptococcus neoformans* and *Cryptococcus gattii* in Tunisia and review of published studies on environmental isolations in Africa. Mycopathologia. 2011;171:355–60. PubMed https://doi.org/10.1007/s11046-010-9381-7

41. Springer DJ, Billmyre RB, Filler EE, Voelz K, Pursall R, Mieczkowski PA, et al. *Cryptococcus gattii* VGIII isolates causing infections in HIV/AIDS patients in Southern California: identification of the local environmental source as arboreal. PLoS Pathog. 2014;10:e1004285. PubMed https://doi.org/10.1371/journal.ppat.1004285

42. Chen Y, Litvintseva AP, Frazzitta AE, Haverkamp MR, Wang L, Fang C, et al. Comparative analyses of clinical and environmental populations of *Cryptococcus neoformans* in Botswana. Mol Ecol. 2015;24:3559–71. PubMed https://doi.org/10.1111/mec.13260

43. Alves GSB, Freire AKL, Bentes AS, Pinheiro JFD, de Souza JVB, Wanke B, et al. Molecular typing of environmental *Cryptococcus neoformans*/*C. gattii* species complex isolates from Manaus, Amazonas, Brazil. Mycoses. 2016;59:509–15. PubMed https://doi.org/10.1111/myc.12499

44. Kangogo M, Bader O, Boga H, Wanyoike W, Folba C, Worasilchai N, et al. Molecular types of *Cryptococcus gattii*/*Cryptococcus neoformans* species complex from clinical and environmental sources in Nairobi, Kenya. Mycoses. 2015;58:665–70. PubMed https://doi.org/10.1111/myc.12411

45. Colom MF, Hagen F, Gonzalez A, Mellado A, Morera N, Linares C, et al. *Ceratonia siliqua* (carob) trees as natural habitat and source of infection by *Cryptococcus gattii* in the Mediterranean environment. Med Mycol. 2012;50:67–73. PubMed https://doi.org/10.3109/13693786.2011.574239

46. Hagen F, Chowdhary A, Prakash A, Yntema J-B, Meis JF. Molecular characterization of *Cryptococcus gattii* genotype AFLP6/VGII isolated from woody debris of divi-divi (*Caesalpinia coriaria*), Bonaire, Dutch Caribbean. Rev Iberoam Micol. 2014;31:193–6. PubMed https://doi.org/10.1016/j.riam.2013.10.007

47. Cogliati M, D'Amicis R, Zani A, Montagna MT, Caggiano G, De Giglio O, et al. Environmental distribution of *Cryptococcus neoformans* and *C. gattii* around the Mediterranean basin. FEMS Yeast Res. 2016;16:fow045. PubMed https://doi.org/10.1093/femsyr/fow045

48. Linares C, Colom MF, Torreblanca M, Esteban V, Romera Á, Hagen F. Environmental sampling of *Ceratonia siliqua* (carob) trees in Spain reveals the presence of the rare *Cryptococcus gattii* genotype AFLP7/VGIV. Rev Iberoam Micol. 2015;32:269–72. PubMed https://doi.org/10.1016/j.riam.2014.11.002

49. Brito-Santos F, Barbosa GG, Trilles L, Nishikawa MM, Wanke B, Meyer W, et al. Environmental isolation of *Cryptococcus gattii* VGII from indoor dust from typical wooden houses in the deep Amazonas of the Rio Negro basin. PLoS One. 2015;10:e0115866. PubMed https://doi.org/10.1371/journal.pone.0115866

50. Khayhan K, Hagen F, Norkaew T, Puengchan T, Boekhout T, Sriburee P. Isolation of *Cryptococcus gattii* from a *Castanopsis argyrophylla* tree hollow (Mai-Kaw), Chiang Mai, Thailand. Mycopathologia. 2017;182:365–70. PubMed https://doi.org/10.1007/s11046-016-0067-7

51. Hagen F, Khayhan K, Theelen B, Kolecka A, Polacheck I, Sionov E, et al. Recognition of seven species in the *Cryptococcus gattii*/*Cryptococcus neoformans* species complex. Fungal Genet Biol. 2015;78:16–48. PubMed https://doi.org/10.1016/j.fgb.2015.02.009

52. Nordlohne E. The Netherlands Antilles. Revista Geografica. 1958;23:103–10.

53. Peel MC, Finlayson BL, McMahon TA. Updated world map of the Köppen-Geiger climate classification. Hydrol Earth Syst Sci. 2007;11:1633–44. https://doi.org/10.5194/hess-11-1633-2007

Appendix Table. Unique georeferenced global environmental isolations of *Cryptococcus gattii* (n = 83) listed in chronologic order, 1989–2016*

Country	Area	Sample year(s)	Longitude	Latitude	Earliest year	Genotype	Mating type	Species	With ±40° limits	With ±35° limits	KG label	KG limits	Ref.
Australia	Balranald, NSW	1989	143.5633	–34.6394	1989	–	–	–	ST	ST	BSk	AR	(*4*)
Australia	Balranald, NSW	1989–1990	143.5633	–34.6394	1989	AFLP4B/VGIb	–	*C. gattii* s.s.	ST	ST	BSk	AR	(*5*)
Australia	Balranald, NSW	1989–1996	143.5633	–34.6394	1989	AFLP4/VGI	α, **a**	*C. gattii* s.s.	ST	ST	BSk	AR	(*6*)
Australia	Hay, NSW	1989	144.5803	–34.1573	1989	–	–	–	ST	ST	BSk	AR	(*4*)
Australia	Hay, NSW	1989–1990	144.5803	–34.1573	1989	AFLP4/VGI	α	*C. gattii* s.s.	ST	ST	BSk	AR	(*6*)
Australia	Barossa Valley (Barossa Reservoir and Nuriootpa), SA	1989	138.95	–34.5333	1989	–	–	–	ST	ST	Csa	TM	(*4*)
Australia	Barossa Valley, SA	1989–1992	138.95	–34.5333	1989	AFLP4A/VGIa	–	*C. gattii* s.s.	ST	ST	Csa	TM	(*5*)
United States	Fort Point, San Francisco, California	1990	–122.477	37.81022	1990	AFLP6A/VGIIa	–	*C. deuterogattii*	ST	TM	Csb	TM	(*7*)
Australia	Tocumwal, NSW	1991	145.569	–35.8122	1991	AFLP4/VGI	–	*C. gattii* s.s.	ST	TM	BSk	AR	(*5*)
Australia	Currumbin, Gold Coast, QLD	1991	153.4667	–28.1333	1991	–	–	–	ST	ST	Cfa	TM	(*8*)
Australia	Currumbin, Gold Coast, QLD	1991–1993	153.4667	–28.1333	1991	AFLP4/VGI	–	*C. gattii* s.s.	ST	ST	Cfa	TM	(*5*)
Australia	Mt. Annan, Greater Sydney, NSW	1991	150.7598	–34.0529	1991	–	–	–	ST	ST	Cfb	TM	(*8*)
Australia	Mt. Annan, Greater Sydney, NSW	1991–1994	150.7598	–34.0529	1991	AFLP4/VGI	–	*C. gattii* s.s.	ST	ST	Cfb	TM	(*5*)
Australia	Busselton, WA	1993	115.3708	–33.6848	1993	AFLP6/VGII	–	*C. deuterogattii*	ST	ST	Csb	TM	(*5*)
Brazil	Rio de Janeiro	<1993	–43.16667	–22.9	1993	AFLP4/VGI	–	*C. gattii* s.s.	T	T	Am	T	(*9*)
Brazil	Teresina	1993	–42.8485	–5.30175	1993	AFLP6/VGII	α	*C. deuterogattii*	T	T	Aw	T	(*10*)
Brazil	Teresina	1993–1997	–42.8485	–5.30175	1993	AFLP6/VGII	α	*C. deuterogattii*	T	T	Aw	T	(*11*)
India	Ferozepur	1995–1996	74.56	30.89	1995	–	–	–	ST	ST	BSh	AR	(*12*)
Australia	Gold Coast, QLD	1996	153.4309	–28.0003	1996	AFLP4/VGI	α	*C. gattii* s.s.	ST	ST	Cfa	TM	(*6*)
Australia	Adelaide, SA	1996	138.5986	–34.9287	1996	AFLP4/VGI	α	*C. gattii* s.s.	ST	ST	Csa	TM	(*6*)
Brazil	Ibirapuera Park, Sao Paulo	1996–1997	–46.6581	–23.6022	1996	–	–	–	ST	ST	Cfa	TM	(*13*)
Italy	Apulia	1996	16.619	40.985	1996	–	–	–	TM	TM	Cfa	TM	(*14*)
United States	San Diego Zoo area, San Diego, California	<1996	–117.157	32.71533	1996	AFLP4/VGI<br>AFLP5/VGIII	–	*C. gattii* s.s.<br>*C. bacillisporus*	ST	ST	BSk	AR	(*5*)
Australia	Coffs Harbour, NSW	1997–2000	153.10	–30.30	1997	–	α	–	ST	ST	Cfa	TM	(*15*)
Australia	Port Macquarie, NSW	1997–2000	152.92	–31.44	1997	–	α	–	ST	ST	Cfa	TM	(*15*)
Australia	St. Ives, Sydney, NSW	1997–1998	151.1667	–33.7333	1997	AFLP4/VGI	α	*C. gattii* s.s.	ST	ST	Cfa	TM	(*6*)
Australia	Eastern Sydney, NSW	1997–2000	151.20	–33.86	1997	–	α, **a**	–	ST	ST	Cfa	TM	(*15*)
Colombia	Cúcuta	1997	–72.5	7.9	1997	–	–	–	T	T	Am	T	(*16*)
Australia	Renmark, SA	1998	140.747	–34.177	1998	AFLP4/VGI	α, **a**	*C. gattii* s.s.	ST	ST	BSk	AR	(*6*)
Australia	Breeza, NSW	1998	150.4667	–31.25	1998	AFLP4/VGI	α	*C. gattii* s.s.	ST	ST	Cfa	TM	(*6*)
Australia	Pilliga, NSW	1998	148.9	–30.35	1998	AFLP4/VGI	α	*C. gattii* s.s.	ST	ST	Cfa	TM	(*6*)
Australia	Port Macquarie, NSW	1998	152.9167	–31.4167	1998	AFLP4/VGI	α	*C. gattii* s.s.	ST	ST	Cfa	TM	(*6*)
Brazil	Ilha de Maracá	1998–1999	–61.6667	3.41667	1998	AFLP6/VGII	**a**	*C. deuterogattii*	T	T	Am	T	(*17*)
Egypt	Qutur and Tanta areas, Gharbia Governorate	1998	30.95614	30.97225	1998	–	–	–	ST	ST	BWh	AR	(*18*)
India	Delhi (northwestern India)	1999–2000	77.22897	28.65381	1999	–	–	–	ST	ST	BSh	AR	(*19*)
Mexico	Mexico City	<1999	–99.1277	19.42847	1999	–	–	–	T	T	Cwb	TM	(*20*)
Australia	Glenbrook, Blue Mountains National Park, Sydney	2000	150.4731	–33.6562	2000	AFLP4/VGI	α	*C. gattii* s.s.	ST	ST	Cfb	TM	(*21*)
Argentina	Buenos Aires City	2001	–58.3772	–34.6131	2001	–	–	–	ST	ST	Cfa	TM	(*22*)

Country	Area	Sample year(s)	Longitude	Latitude	Earliest year	Genotype	Mating type	Species	With ±40° limits	With ±35° limits	KG label	KG limits	Ref.
Argentina	Rep. de Chile Park, de los Patricios Park, Centenario Park, and N. Avellaneda Park in Buenos Aires City	2002	–58.3772	–34.6131	2002	AFLP4/VGI	–	*C. gattii* s.s.	ST	ST	Cfa	TM	(*23*)
Canada	Cameron Lake, B.C.	2002	–124.619	49.29133	2002	AFLP6A/VGIIa	–	*C. deuterogattii*	TM	TM	Csb	TM	(*24*)
Canada	Courtenay, B.C.	2002	–124.994	49.68657	2002	AFLP6A/VGIIa	–	*C. deuterogattii*	TM	TM	Csb	TM	(*24*)
Canada	Duncan, B.C.	2002	–123.703	48.78293	2002	AFLP4/VGI<br>AFLP6B/VGIIb	–	*C. gattii* s.s.<br>*C. deuterogattii*	TM	TM	Csb	TM	(*24*)
Canada	MacMillan Park, Cathedral Grove, B.C.	2002	–124.669	49.28293	2002	AFLP6A/VGIIa	α	*C. deuterogattii*	TM	TM	Csb	TM	(*25*)
Canada	Nanaimo, B.C.	2002	–123.936	49.16634	2002	AFLP6A/VGIIa	–	*C. deuterogattii*	TM	TM	Csb	TM	(*24*)
Canada	Parksville, B.C.	2002	–124.314	49.32	2002	AFLP6A/VGIIa	–	*C. deuterogattii*	TM	TM	Csb	TM	(*24*)
Canada	Port Alberni, B.C.	2002	–124.808	49.234	2002	AFLP6A/VGIIa,<br>AFLP6B/VGIIb	–	*C. deuterogattii*	TM	TM	Csb	TM	(*24*)
Canada	Rathtrevor Beach Provincial Park, Parksville, B.C.	2002	–124.266	49.32096	2002	AFLP6A/VGIIa,<br>AFLP6B/VGIIb	α	*C. deuterogattii*	TM	TM	Csb	TM	(*25*)
Canada	Victoria, B.C.	2002	–123.369	48.43294	2002	AFLP6A/VGIIa	–	*C. deuterogattii*	TM	TM	Csb	TM	(*24*)
Colombia	Cali	2002–2003	–75.68333	5.0	2002	AFLP6/VGII	**a**	*C. deuterogattii*	T	T	Af	T	(*26*)
Colombia	Cúcuta	2002–2003	–72.01667	6.933333	2002	AFLP4/VGI<br>AFLP5/VGIII<br>VGIV (AFLP type not specified)	α	*C. gattii* s.s.<br>*C. bacillisporus*<br>–	T	T	Cfb	TM	(*26*)
Colombia	Bogota	2002–2003	–74.08333	4.6	2002	AFLP6/VGII	**a**	*C. deuterogattii*	T	T	Cfb	TM	(*26*)
Colombia	Cundinamarca	2002–2003	–73.05	3.666667	2002	AFLP6/VGII<br>VGIV (AFLP type not specified)	**a**<br>α	*C. deuterogattii*<br>–	T	T	Am	T	(*26*)
Colombia	Medellin	2002–2003	–73.88333	5.433333	2002	AFLP6/VGII	**a**	*C. deuterogattii*	T	T	Cfb	TM	(*26*)
India	Jabalpur City (Central India)	2002–2004	79.95006	23.16697	2002	–	–	–	T	T	Csa	TM	(*27*)
United States	Lynden, Washington	2002–2006	–122.452	48.9465	2002	AFLP6A/VGIIa	–	*C. deuterogattii*	TM	TM	Cfb	TM	(*24*)
Australia	Mt. Druitt, Sydney, NSW	<2003	150.8167	–33.7667	2003	AFLP4/VGI<br>AFLP6/VGII	α<br>α	*C. gattii* s.s.<br>*C. deuterogattii*	ST	ST	Cfa	TM	(*28*)
Colombia	La Calera	2003	–73.9693	4.72069	2003	–	**a**	–	T	T	Cfb	TM	(*29*)
Canada	Saltspring Island, B.C.	2004–2005	–123.408	48.769	2004	AFLP4/VGI	**–**	*C. gattii* s.s.	TM	TM	Csb	TM	(*30*)
Canada	Hornby Island	2005	–124.667	49.525	2005	AFLP6A/VGIIa	**–**	*C. deuterogattii*	TM	TM	Csb	TM	(*24*)
Argentina	Resistencia, Chaco	2006	–58.9839	–27.4606	2006	AFLP5/VGIII	–	*C. bacillisporus*	ST	ST	Cfa	TM	(*31*)
Canada	Elk Falls Provincial Park, Campbell River	2006	–125.251	50.026	2006	–	–	–	TM	TM	Cfb	TM	(*24*)
Argentina	Parque España, La Paz, Entre Rios	2007	–59.6434	–30.7437	2007	AFLP4/VGI	–	*C. gattii* s.s.	ST	ST	Cfa	TM	(*31*)
Argentina	Rosario, Santa Fe	2007	–60.6393	–32.9468	2007	AFLP4/VGI	–	*C. gattii* s.s.	ST	ST	Cfa	TM	(*31*)
Brazil	Botafogo district, Rio de Janeiro	2008–2010	–43.1856	–22.969	2008	AFLP4/VGI	–	*C. gattii* s.s.	T	T	Am	T	(*32*)
Colombia	Cúcuta	2008–2009	–72.51	7.913333	2008	AFLP4/VGI<br>AFLP5/VGIII	**a**<br>α	*C. gattii* s.s.<br>*C. bacillisporus*	T	T	Cfb	TM	(*33*)
Brazil	Belem, Para	<2009	–48.50417	–1.45556	2009	AFLP6/VGII	–	*C. deuterogattii*	T	T	Am	T	(*34*)
India	Guindy National Park, Chennai, South India	<2009	80.21941	12.99668	2009	–	–	–	T	T	Aw	T	(*35*)

Country	Area	Sample year(s)	Longitude	Latitude	Earliest year	Genotype	Mating type	Species	With ±40° limits	With ±35° limits	KG label	KG limits	Ref.
Italy	Reggio Calabria (southern Italy)	2009	15.65	38.07	2009	AFLP4/VGI	α	*C. gattii* s.s.	ST	TM	Csa	TM	(*36*)
Puerto Rico	Guanica Dry Forest	<2010	–66.908	17.97163	2010	AFLP6/VGII<br>AFLP7/VGIV	α<br>α	*C. deuterogattii*<br>*C. tetragattii*	T	T	Aw	T	(*37*)
United States	Near Silver Falls State Park, Oregon (based on Figure 1 in main text)	2010–2011	–122.6336	44.90544	2010	AFLP4/VGI, AFLP6A/VGIIa, AFLP6B/VGIIb, AFLP6C/VGIIc	–	*C. gattii* s.s., *C. deuterogattii*	TM	TM	Csb	TM	(*38*)
The Netherlands	Berg en Dal	2011	5.916667	51.82167	2011	AFLP4/VGI	α	*C. gattii* s.s.	TM	TM	Cfb	TM	(*39*)
Tunisia	Sfax region (southern Tunisia)	<2011	10.76028	34.74056	2011	–	α	–	ST	ST	BSh	AR	(*40*)
United States	Los Angeles, California	2011–2012	–118.244	34.05223	2011	AFLP4/VGI<br>AFLP5A/VGIIIa<br>AFLP5B/VGIIIb	α<br>α<br>**a,** α	*C. gattii* s.s.	ST	ST	Csb	TM	(*41*)
Botswana	Francistown	2012	27.50788	–21.17	2012	–	**a,** α	–	T	T	BSh	AR	(*42*)
	Gaborone	2012	25.933	–24.651	2012	–	α	–	ST	ST	BSh	AR	
	Maun	2012	23.415	–20.007	2012	–	**a,** α	–	T	T	BSh	AR	
Brazil	Manaus	2012–2014	–60.052	–3.109	2012	AFLP6/VGII	α	*C. deuterogattii*	T	T	Af	T	(*43*)
Kenya	Nairobi	2012–2013	36.83333	–1.28333	2012	AFLP4/VGI	–	*C. gattii* s.s.	T	T	Cfb	TM	(*44*)
Spain	Alicante	<2012	–0.48149	38.34517	2012	AFLP4/VGI	α	*C. gattii* s.s.	ST	TM	BSk	AR	(*45*)
Spain	Barcelona	<2012	2.158987	41.38879	2012	AFLP4/VGI	α	*C. gattii* s.s.	TM	TM	Csa	TM	(*45*)
Bonaire	Lagun Goto, Rincon village, Hato village	2013	–68.3258	12.21501	2013	AFLP6/VGII	α	*C. deuterogattii*	T	T	BSh†	AR	(*46*)
Greece	Athens	2013	23.71622	37.97945	2013	AFLP4/VGI	α	*C. gattii* s.s.	ST	TM	Csa	TM	(*47*)
Greece	Salamina Island	2013	23.50677	37.92189	2013	AFLP4/VGI	α	*C. gattii* s.s.	ST	TM	Csa	TM	(*47*)
Italy	Ragalna, Catania	2013	15.08233	37.43505	2013	AFLP4/VGI	α	*C. gattii* s.s.	ST	TM	Csa	TM	(*47*)
Italy	Route Brindisi-Fasano	2013	17.69123	40.60921	2013	AFLP4/VGI	**a**	*C. gattii* s.s.	TM	TM	Csa	TM	(*47*)
Italy	Route Gallipoli-Collepasso	2013	17.99538	40.0559	2013	AFLP4/VGI	**a**	*C. gattii* s.s.	TM	TM	Csa	TM	(*47*)
Spain	El Perello, Tarragona	2013	0.71335	40.87642	2013	AFLP7/VGIV	α	*C. tetragattii*	TM	TM	Csa	TM	(*47*, *48*)
Spain	Campello, Alicante	2014	–0.398	38.429	2014	AFLP4/VGI	α	*C. gattii* s.s.	ST	TM	BSk	AR	(*47*)
Spain	Mendivil, Navarra	2014	–1.451	42.66366	2014	AFLP4/VGI	α	*C. gattii* s.s.	TM	TM	Cfb	TM	(*47*)
Brazil	Santa Isabel do Rio Negro, Amazonas state	<2015	–65.01889	–0.41417	2015	AFLP6/VGII	α, **a**	*C. deuterogattii*	T	T	Af	T	(*49*)
Thailand	Queen Sirikit Botanic Garden, Chiang Mai Province	<2016	98.86	18.89953	2016	AFLP4/VGI	–	*C. gattii* s.s.	T	T	Aw	T	(*50*)

*For longitude, positive numbers refer to degrees east of the prime meridian and negative numbers refer to degrees west of the prime meridian. For latitude, positive numbers refer to degrees north of the equator and negative numbers refer to degrees south of the equator. The latitudinal boundaries of ±40° and ±35° refer to degrees north (+) and south (-) of the equator. For each isolation, we recorded the country and area of isolation, the range of years when the isolations were collected (if the earliest year yielding positive isolations was provided, this would be the start of the range), the coordinates where the isolation was found, the earliest possible year of collection recorded for this analysis, and the climate classifications for each isolation, as designated by solar definitions with ±40° and ±35° limits as well as by the KG system. Genotype (including subtype), mating type, and species (*51*) are also provided, if specified by the study. It is important to note that some studies have only used phenotypic methods (e.g., CGB agar) to differentiate *C. gattii* sensu lato from *C. neoformans* s.l.. This is, however, not always a reliable method. Thus, data from this list need to be interpreted with caution. AR, arid; B.C., British Columbia; KG, Köppen-Geiger; NSW, New South Wales; QLD, Queensland; ref., reference; SA, South Australia; ST, subtropical; T, tropical; TM, temperate; WA, Western Australia.

†The KG system does not classify the island of Bonaire (Dutch Caribbean) in its map of observed climates during 1976–2000. We, therefore, obtained the classification of Bonaire from (*52*) in addition to our own categorization using the KG climate classification criteria (*53*) and present-day climate averages of the island (https://www.ncdc.noaa.gov/).

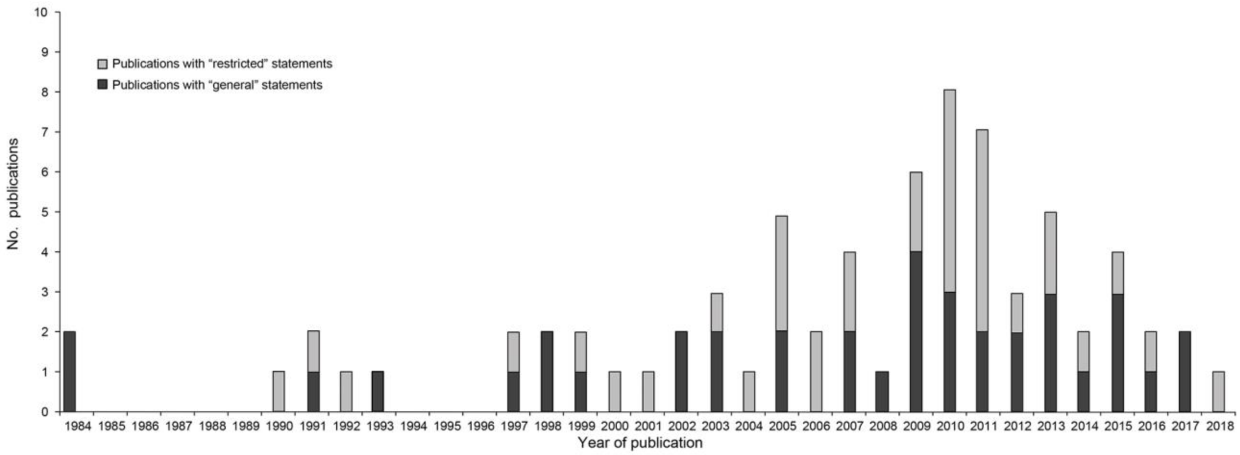

Appendix Figure. Publications from 1984 through November 2018 discussing pre-1999 areas of endemicity of *Cryptococcus gattii* (n = 73). Publications containing statements claiming *C. gattii* was strictly found in tropical and subtropical areas before the 1999 Vancouver Island outbreak were labeled as "restricted" (n = 35), while those containing statements that also acknowledged other areas of endemicity before the 1999 Vancouver Island outbreak were labeled as "general" (n = 38).
